# Generation of Two-Line Restorer Line with Low Chalkiness Using Knockout of *Chalk5* through CRISPR/Cas9 Editing

**DOI:** 10.3390/biology13080617

**Published:** 2024-08-15

**Authors:** Gucheng Fan, Jiefeng Jiang, Yu Long, Run Wang, Famao Liang, Haiyang Liu, Junying Xu, Xianjin Qiu, Zhixin Li

**Affiliations:** 1 Engineering Research Center of Ecology and Agricultural Use of Wetland, Ministry of Education, College of Agriculture, Yangtze University, Jingzhou 434025, China; fangucheng123@outlook.com (G.F.); m18162389088@163.com (Y.L.); 13217213017@163.com (R.W.); hyliu@yangtzeu.edu.cn (H.L.); jyxu@yangtzeu.edu.cn (J.X.); 2 MARA Key Laboratory of Sustainable Crop Production in the Middle Researches of the Yangtze River (Co-Construction by Ministry and Province), College of Agriculture, Yangtze University, Jingzhou 434025, China; 3 Ningbo Academy of Agricultural Science, Ningbo 315101, China; jiefjiang@126.com; 4 Yichang Academy of Agricultural Sciences, Yichang 443004, China; liangfamao92@hotmail.com

**Keywords:** rice, 9311, chalkiness, gene editing, *Chalk5*

## Abstract

**Simple Summary:**

*Chalk5* is an important gene used in rice breeding to improve rice chalkiness, which greatly affects rice quality. For this study, the first exon of *Chalk5* was edited, and two different knockout mutants were obtained. These two lines showed a decreased percentage of grains with chalkiness, chalkiness degree, and seed setting ratio. Moreover, their chalkiness was insensitive to temperature during the grain-filling stage, and the head milled rice rate was improved even under high-temperature conditions. The two lines showed significantly reduced PGWC and DEC without changes in other agronomic traits in the hybrid background. These results indicate that *Chalk5* gene-edited lines are useful in *indica* hybrid rice breeding for a high yield and superior grain quality in high-temperature growing environments.

**Abstract:**

Chalkiness is an important grain quality trait in rice. *Chalk5*, encoding a vacuolar H^+^-translocating pyrophosphatase, is a major gene affecting both the percentage of grains with chalkiness (PGWC) and chalkiness degree (DEC) in rice. Reducing its expression can decrease both PGEC and DEC. In this study, the first exon of *Chalk5* was edited in the elite restorer line 9311 using the CRISPR/Cas9 system and two knockout mutants were obtained, one of which did not contain the exogenous Cas9 cassette. PGWC and DEC were both significantly reduced in both mutants, while the seed setting ratio (SSR) was also significantly decreased. Staggered sowing experiments showed that the chalkiness of the mutants was insensitive to temperature during the grain-filling stage, and the head milled rice rate (HMRR) could be improved even under high-temperature conditions. Finally, in the hybrid background, the mutants showed significantly reduced PGWC and DEC without changes in other agronomic traits. The results provide important germplasm and allele resources for breeding high-yield rice varieties with superior quality, especially for high-yield *indica* hybrid rice varieties with superior quality in high-temperature conditions.

## 1. Introduction

Rice is one of the most important staple crops and is a carbohydrate source for more than half of the world’s population [1]. In Asia, it is the staple food for about 90% of the population. With the improvement in living standards, people are paying increasing attention to high-quality rice [2]. Thus, superior quality has become a key breeding target that is equal to a high yield. Rice quality parameters include milling quality, appearance quality, cooking and eating quality, and nutritional quality. Among them, chalkiness is an important appearance quality trait that directly affects the market value of rice. Chalkiness results from incomplete filling during grain development [3], and can be classified as white belly, white core, and white back. It is usually measured using the percentage of grains with chalkiness (PGWC) and chalkiness degree (DEC). High chalkiness not only reduces the head milled rice yield, but also affects palatability. Thus, breeding rice varieties with low chalkiness is crucial to meet the demand for superior quality rice.

Chalkiness is a typical quantitative trait controlled by multiple genes [4]. In recent years, researchers have identified numerous quantitative trait loci (QTLs) affecting chalkiness and have cloned several genes using various genetic populations and different strategies [2,4,5,6,7,8,9,10,11,12,13,14,15,16,17,18]. Among them, *Chalk5* is a major gene controlling both PGWC and DEC, which has been cloned using a population derived from Zhenshan97 with high chalkiness and H94 with low chalkiness [19]. *Chalk5* encodes a vacuolar H^+^-translocating pyrophosphatase, which affects protein body formation and vesicle-like structure accumulation through disrupting the pH homeostasis of the endomembrane system during seed development, leading to the formation of air spaces in the endosperm and ultimately causing chalkiness. The expression level of *Chalk5* is significantly correlated with the degree of chalkiness, and a higher expression results in more chalkiness. A haplotype analysis showed that two SNPs (C/T and A/T) at 721 and 485 upstream of the start codon of *Chalk5* were significantly associated with grain chalkiness.

*Chalk5* is an important gene for improving rice appearance quality in molecular breeding. The introgression of the low-chalkiness *indica* allele of *Chalk5* significantly reduced chalkiness and further optimized the milling quality of *indica–japonica* hybrid rice [20]. Moreover, different combinations of alleles of *GS3*, *Chalk5*, and *Chalk7* had large effects on grain chalkiness [21]. A favorable allele combination of the three genes could greatly reduce rice chalkiness without negatively affecting the grain yield.

In addition to genetic factors, the temperature during the grain-filling stage also significantly affects chalkiness in rice [22]; in particular, the temperature during the initial 15 days of grain filling has a significant impact on chalkiness. When the average temperature exceeds 26 °C, chalkiness increases significantly. Under high-temperature conditions, the expression of *Chalk5* increases, while the expression of starch synthesis-related genes decreases and the expression of sucrose and starch degradation-related genes increases, leading to reduced accumulation of sucrose and starch and ultimately resulting in chalkiness [23].

CRISPR/Cas9 is an effective gene editing technique that has emerged in recent years, which can precisely modify target genes to create new mutations [24]. Compared to marker-assisted breeding, CRISPR/Cas9 can precisely modify target genes and avoid negative effects through the introduction of other negative genes into the background; therefore, it has wide application prospects in functional studies and crop improvement. For example, *Chalk5* is closely linked to the genes *GS5* and *qSW5* that control grain width [25,26,27]. This linkage of different genes results in a positive correlation between chalkiness and grain width. However, if we want to decrease chalkiness, the reduction in grain width will lead to a decrease in thousand grain weight, ultimately affecting the rice yield. Therefore, editing *Chalk5* using the CRISPR/Cas9 system can improve quality without reducing the yield.

The functional divergency of *Chalk5* in cultivars is mainly caused by the polymorphism sites in the promoter. A strong functional allele of *Chalk5* is highly expressed at the mRNA level with high chalkiness. A recent study editing the promoter of *Chalk5* using this CRISPR/Cas9 system also validated it [28]. The expression of *Chalk5* is suppressed with considerable effect, but whether the knockout of *Chalk5* would produce a more valuable effect for high-quality rice breeding is still unknown. In this study, we used the CRISPR/Cas9 system to edit the first exon of *Chalk5* in the backbone restorer line 9311, and we evaluated the yield and chalkiness of the knockout lines under different filling temperatures to assess their sensitivity to the temperature. Further, yield and quality traits were evaluated in the hybrid backgrounds through crossing the gene editing lines with two-line sterile lines. The results provide important germplasm and allele resources for breeding high-yield rice varieties with superior quality, especially for high yields with superior quality *indica* hybrid rice in high-temperature conditions.

## 2. Materials and Methods

### 2.1. Materials

The elite two-line restorer line 9311 was used as the transgenic recipient and the control. In addition, hybrid combinations were made between two photo–thermo-sensitive genic male sterile lines, LX03S and Huhan82S, and the edited lines. LX03S was bred by our group and Huhan82S was introduced from the Shanghai Agrobiological Gene Center, China.

### 2.2. Gene Editing

Based on the *Chalk5* sequence provided by the TIGR database (http://rice.plantbiology.msu.edu/, accessed on 10 November 2022), a 20 bp target site was designed in the first exon of *Chalk5*, with the sequence ATGACGGAGTACAAGTACCT and with the protospacer adjacent motif “GGG” according to a previous study [29]. The ligation reaction conditions were 37 °C for 20 min, 5 cycles; 37 °C for 10 min, 20 °C for 10 min, 50 cycles; 37 °C for 20 min; and 80 °C for 5 min. The successfully ligated products were transformed into chemically competent *E. coli* cells using heat shock, and the transformed *E. coli* cells were plated on LB solid medium containing kanamycin to screen for positive colonies. The plasmids extracted from the positive colonies were introduced into EHA105 *Agrobacterium tumefaciens* competent cells, and the constructed pBWA(V)H_cas9i2-*Chalk5* plasmid was then transformed into the rice materials via Agrobacterium-mediated transformation, with positive mutant lines selected using hygromycin. The gene editing in this experiment was finished by Wuhan BioRun Bioscience Co., Ltd. (Wuhan, China).

After obtaining the transgenic seedlings, the mutation sites were identified using sequencing. Leaf samples were collected, and DNA was extracted using the CTAB method [4]. The PCR amplification and sequencing were performed according to the method described by Qiu et al. [30], using the primers F: 5′-CGCCGTGCTCCAGTGGTAC and R: 5′-TGAACGCCGCGTTCGCCAG. The primers F: 5′-ATCCAGCACGCCATCTCCGTTGG and R: 5′-CGTAGTAGAGCCCGAACGCGTTCA were used to detect whether the vector in homozygous mutants was present.

### 2.3. Field Experiment and Trait Evaluation

In summer 2020, three T_0_-edited seedlings were planted at the Yangtze University experimental farm in Jingzhou (30.18° E, 112.15° E), Hubei. After a sequencing analysis, seeds from two positive plants were harvested. In winter 2020, 20 T_1_ plants per line were planted in a phytotron at Yangtze University. After a sequencing analysis and phenotypic evaluation, two homozygous plants with agronomic traits similar to 9311 were selected, and their seeds were harvested. In summer 2021, two T_2_ lines (TC1 and TC5) were planted in the Yangtze University experimental field. Materials were sowed on 15 May, and each line was planted in 5 rows with 8 plants per row at a spacing of 20 cm × 20 cm. Field management followed standard practices.

After maturity, single-plant harvesting was conducted, and 10 plants with identical growth were selected to evaluate the panicle number (PN), spikelet number per panicle (SN), filled grain number per panicle (GN), seed setting ratio (SSR), thousand grain weight (KGW), and grain yield (GY). After a yield evaluation, seeds from two plants were mixed and stored at room temperature for 3 months before evaluating the milling quality and appearance quality, including brown rice rate (BRR), milled rice rate (MRR), head milled rice rate (HMRR), grain length (GL), grain width (GW), length-to-width ratio (LWR), percentage of grains with chalkiness (PGWC), and chalkiness degree (DEC). These traits were measured as described by Qiu et al. [9,31].

In 2022, the original 9311 and the two edited lines were sown in four staggered sowings, with an interval of 15 days between each sowing, starting from 27 April with two replicates for each batch of sowing. The remaining planting methods were the same as in summer 2021. The heading date of each line and the temperatures during the first 15 days of grain filling were recorded. After the seeds were fully matured, two plants from each line were randomly mix-harvested. After storing at room temperature for 3 months, the HMRR, PGWC, and DEC were measured.

Furthermore, in summer 2021, 9311 and TC1 (Cas9 backbone free) were test-crossed with LX03S and Huhan82S, respectively. In summer 2022, the generated F1 hybrids were planted at the Yangtze University experimental farm in Jingzhou, Hubei. They were seeding on 27 April, following the same methods as in summer 2021. After maturity, 10 plants with identical growth were selected from each line to evaluate the PN, SN, GN, SSR, KGW, and GY values. Seeds from two plants were mixed and stored at room temperature for 3 months and then measured for the HMRR, PGWC, and DEC values.

### 2.4. Data Analysis

The raw data were organized using Excel 2016, and a statistical description and *t*-tests were performed using the Statistica 5.5 software [4].

## 3. Results

### 3.1. Generation of Chalk5 Knockout Homozygous Mutants

**Figure 1:**
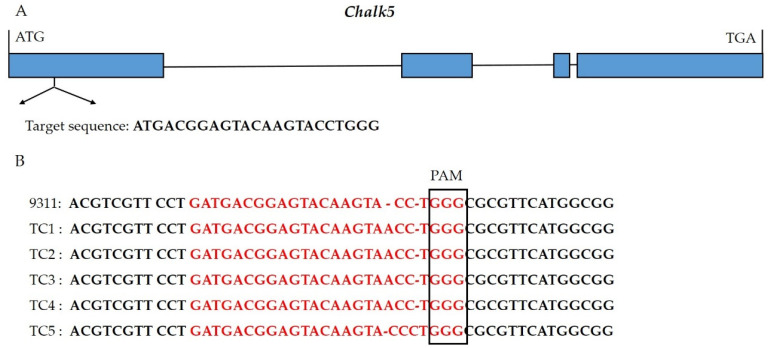
Identification and generation of the knockout lines of *Chalk5*. (**A**) The gene structure of *Chalk5* and position of the target sequences in the first exon. Blue boxes and black lines show the exons and introns, respectively; (**B**) identification of mutants in *Chalk5* using sequencing of the target site in T_1_ transgenic individuals. PAM represents the protospacer adjacent motif. In red, base sequences were target sites.

Using the CRISPR/Cas9 system, the first exon of *Chalk5* was edited in 9311 and three transgenic materials were obtained. Through target site sequencing, two mutants were identified and harvested. The *Chalk5* alleles in the two mutants encoded 98 and 273 amino acids (Supplementary File S1). Two homozygous mutants (TC1 and TC5) with agronomic traits similar to 9311 were selected from T_1_ lines (Figure 1). Among them, TC1 had an inserted A, resulting in the premature termination of amino acid coding. TC5 had an inserted C, leading to a frameshift mutation. Additionally, we performed genotyping using a Cas9-specific marker on the two positive transgenic single plants and found that TC1 did not contain the foreign Cas9 backbone.

### 3.2. Chalk5 Knockout Homozygous Mutants Showed Significantly Reduced Chalkiness

**Figure 2:**
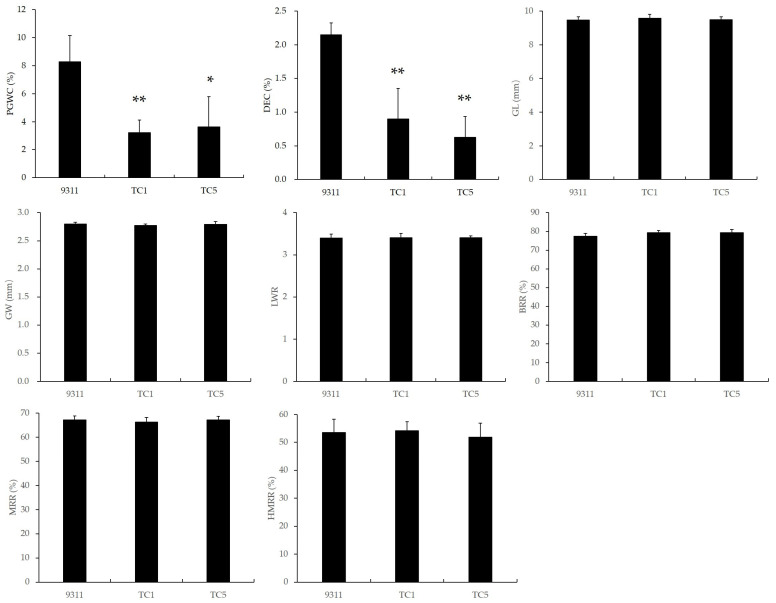
The appearance and milling qualities of two knockout lines of *Chalk5* and 9311. PGWC, percentage of grains with chalkiness; DEC, chalkiness degree; GL, grain length; GW, grain width; LWR, length-to-width ratio; BRR, brown rice rate; MRR, milled rice rate; and HMRR, head milled rice rate. * and ** represent significant level at *p* < 0.05 and 0.01, respectively, when compared to 9311 using a *t*-test. Error bars represent the standard deviation for each line (n = 20).

The milling and appearance quality of the two *Chalk5* knockout homozygous mutants are shown in Figure 2 and Supplementary Figure S1. For chalkiness, the PGWC in the knockout lines was 3.23% and 3.63%, which were significantly reduced from the wild-type (8.28%). The DEC in TC1 and TC5 was only 0.90% and 0.63%, respectively, which was also significantly lower than the wild-type (2.15%). Thus, the knockout of *Chalk5* reduced both the PGWC and DEC values efficiently. The grain shape is an important appearance quality trait and influences chalkiness. In two knockout lines, the GL, GW, and LWR values had no significant difference from the wild-type, and both are typically *indica* slender grain. For milling quality traits, the BRRs were 79.27% and 79.30%, the MRRs were 66.26% and 67.19%, and the HMRRs were 54.15% and 51.83%, which were similar to 9311. These results indicated that the functional loss of *Chalk5* significantly reduced the PGWC and DEC values without affecting other appearance quality and milling quality traits.

The yield-related traits of the two *Chalk5* knockout homozygous mutants are shown in Supplementary Table S1. The PN and KGW values of the mutants were not significantly different from 9311, while the SSR was significantly decreased in mutants. The SN was higher in TC5, and the GN and GY values were significantly decreased only in TC1, but these trait variations were not common characteristics of knockout lines. These results suggested that the functional loss of *Chalk5* led to a decrease in the SSR, but might not affect other yield-related traits.

### 3.3. Chalkiness of Chalk5 Knockout Homozygous Mutants Was Insensitive to Temperature

**Figure 3:**
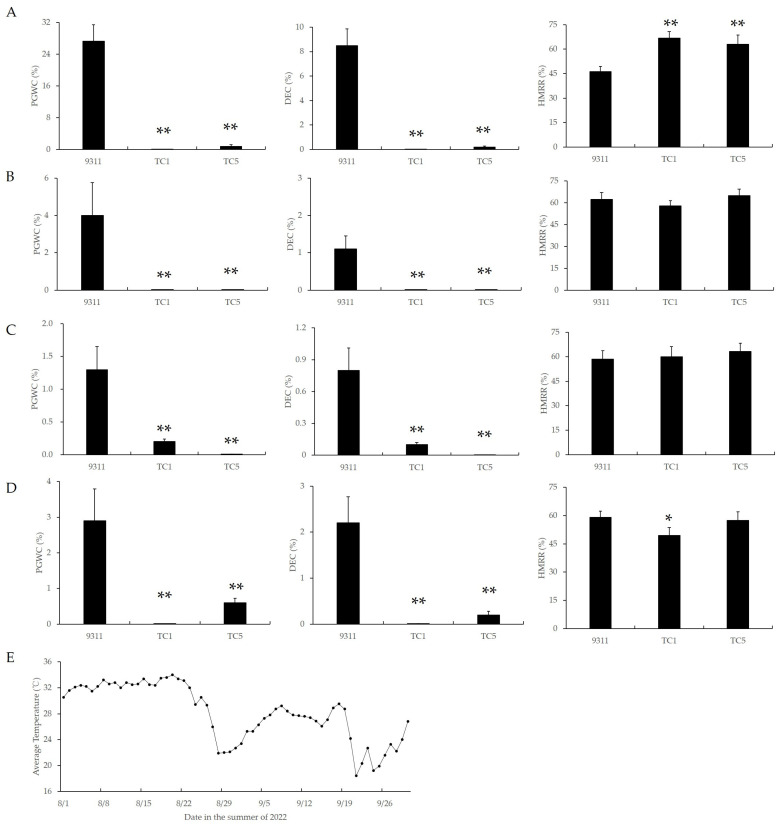
The appearance and milling qualities of two knockout lines of *Chalk5* and 9311 during different sowing stages. (**A**–**D**) PGWC, DEC, and HMRR values detected during the four sowing stages; (**E**) average temperature from 1 August to 30 September in summer 2022. The average temperatures of the first, second, third, and fourth sowing stages were 31.80 °C, 27.22 °C, 27.14 °C, and 25.49 °C during the first 15 days of the grain-filling stage. PGWC, percentage of grains with chalkiness; DEC, chalkiness degree; and HMRR, head milled rice rate. * and ** represent significance at *p* < 0.05 and 0.01, respectively. Error bars represent the standard deviation for each line (n = 20).

To investigate the temperature sensitivity of chalkiness in *Chalk5* knockout homozygous mutants, a staggered sowing experiment was conducted. Four staggered sowings were set with an interval of 15 days between each sowing, starting from 27 April, and their heading dates were about 10 August, 20 August, 29 August, and 8 September. As the sowing date was delayed, the average temperature during the first 15 days of grain filling gradually decreased (Figure 3E). Accordingly, the PGWC and DEC values in 9311 also gradually decreased (Figure 3A–D). In all four sowing periods, the PGWC and DEC values in TC1 were significantly lower than in 9311, and there were no significant differences among different sowing periods within the same mutant. These results indicated that the chalkiness in 9311 was sensitive to temperature during the grain-filling stage, while the chalkiness of the *Chalk5* knockout mutants was insensitive to temperature during this stage, suggesting that the functional loss of *Chalk5* led to temperature insensitivity in chalkiness.

Furthermore, in the first sowing period, the HMRR value in mutants was significantly higher than that of 9311; in the second and third periods, the HMRR value in mutants was similar to 9311; and in the fourth period, the HMRR value in TC1 was slightly reduced (Figure 3; Supplementary Figure S2). These results suggested that the functional loss of *Chalk5* could improve the HMRR value under high-temperature conditions.

### 3.4. Chalk5 Knockout Homozygous Mutants Significantly Decreased Chalkiness in Hybrid Background 

To evaluate the effects of *Chalk5* knockout homozygous mutants on milled rice and the appearance quality and yield traits in the hybrid background, TC1 was selected and crossed with LX03S and Huhan82S. The PGWC and DEC values of LX03S/TC1 and Huhan82S/TC1 were both significantly lower than those of LX03S/9311 and Huhan82S/9311 (Table 1), while other agronomic traits showed no significant differences. Interestingly, the HMRR value of LX03S/TC1 and Huhan82S/TC1 was similar to that of LX03S/9311 and Huhan82S/9311. These results indicated that although the functional loss of *Chalk5* led to a decrease in the SSR and GY values, the hybrid combinations derived from the two-line sterile lines LX03S, Huhan82S, and TC1 showed a similar SSR, thus resulting in almost the same GY value, probably due to a complementary effect between sterile lines and the *Chalk5* knockout homozygous mutants in the hybrid background.

## 4. Discussion

In recent years, people have been paying increasing attention to rice quality. During the past two decades, the DEC value of Chinese-approved *indica* rice varieties has gradually decreased [32]. However, of all the quality traits of the 479 Chinese-approved *indica* hybrid rice varieties, the variance in DEC is the most significant, indicating that chalkiness is becoming an important trait affecting rice quality. Chalkiness is one of the most challenging qualities for hybrid rice, and very limited advances have been made in recent decades using the conventional breeding method. *Chalk5* encodes a vacuolar H^+^-translocating pyrophosphatase that disrupts the pH homeostasis of the endomembrane system during seed development, affecting protein body formation and vesicle-like structure accumulation, leading to the formation of air spaces in the endosperm and causing chalkiness. Transgenic experiments have shown a significant positive correlation between the expression level of *Chalk5* and chalkiness. Therefore, chalkiness can be reduced through decreasing the expression of *Chalk5*. Liu et al. [33] edited *Chalk5* in the *japonica* rice variety Nanjing9108, and found that the mutants had significantly reduced chalkiness. Gann et al. [28] edited the promoter of *Chalk5* in the *japonica* rice variety Nipponbare, and the expression levels of the mutants were significantly decreased, leading to significantly reduced chalkiness. In this study, we used the CRISPR/Cas9 system to edit the first exon of *Chalk5* in the *indica* rice variety 9311 and obtained two types of *Chalk5* homozygous mutants. *Chalk5* encodes a peptide of 768 amino acids in length with an H^+^ PPase domain spanning from the 18th to the 756th amino acid. TC1 had an inserted A, resulting in a premature stop codon and producing a truncated peptide of 98 amino acids, while TC5 had an inserted C, causing a frameshift mutation and producing a truncated peptide of 273 amino acids. Both types of mutation disrupt the H^+^ PPase domain, leading to the functional loss of *Chalk5*. The PGWC value in the mutants was 3.23% and 3.63%, and the DEC value was 0.90% and 0.63%, with almost no chalkiness. These results demonstrated that we could develop a superior quality germplasm with almost no chalkiness through editing the promoter or coding region of *Chalk5* to reduce its expression to extremely low levels or cause a complete loss of function.

Chalkiness is a typical quantitative trait controlled by multiple genes and is easily affected by the environment. A high temperature during grain filling has a significant impact on chalkiness. When the average temperature during the first 15–20 days of grain filling exceeds 26 °C, the PGWC and DEC values increase significantly [22]. In some varieties, the expression of *Chalk5* is induced by a high temperature, leading to increased chalkiness [28]. In this study, we used the CRISPR/Cas9 system to create two types of *Chalk5* loss-of-function mutants, which is equivalent to reducing the expression of *Chalk5* to zero. By using staggered sowing, we allowed 9311 and the mutants to undergo grain filling under different temperature conditions. When the average temperature during the filling stage was 26–32 °C, the PGWC and DEC values in 9311 ranged from 1.30% to 27.32% and 0.83% to 8.49%, respectively, with a noticeable increase when the filling temperature was 32.9 °C. Therefore, the *Chalk5* allele in 9311 is temperature sensitive. In contrast, the PGWC and DEC values in the mutants ranged from 0% to 0.80% and 0% to 0.2%, respectively (Figure 3), without significant differences at different grain-filling temperatures. Thus, the *Chalk5* loss-of-function allele exhibits temperature insensitivity. Moreover, the mutants can improve the HMRR value under high-temperature conditions. The Yangtze River Valley is one of the most important rice production areas in China [34], where high temperatures in summer not only reduce the rice yield but also lead to increased chalkiness and decreased HMRR. As the chalkiness of the mutants is insensitive to temperature during the grain-filling stage and the HMRR value does not decrease at high temperatures, these mutants are important germplasm resources for improving the quality of parental lines in the Yangtze River Valley.

The edited lines showed a significant decrease in the SSR, thus resulting in a significant decrease in the GY value. Therefore, breeders should be aware of the negative impacts of quality improvement on the yield. In the hybrid background, the PGWC and DEC values were significantly reduced compared to 9311, while the SSR showed no significant difference. This means that the sterile line background can compensate for the negative effect of the decreased SSR of the edited line. Therefore, these mutants can serve as important germplasm resources for improving the appearance quality of rice, especially for hybrid varieties without a yield penalty in the Yangtze River Valley, where the temperature is normally high during the grain-filling stage. Most probably, the hybrid combination with a high yield and superior grain quality will be developed using *Chalk5* gene-edited lines.

## 5. Conclusions

Two types of loss-of-function mutants were obtained through editing the first exon of *Chalk5* using the CRISPR/Cas9 system. The PGWC and DEC values, as well as the SSR, were all significantly reduced in the mutants. The PGWC and DEC values in the mutants were insensitive to high temperatures during the grain-filling stage, and the HMRR value was significantly improved under high-temperature conditions. A hybrid combination showed a significant decrease in PGWC and DEC values, while its yield-related traits showed no significant changes. These results indicate that *Chalk5* gene-edited lines are useful in *indica* hybrid rice breeding for high yields and superior grain quality in high-temperature growing environments.

## Figures and Tables

**Table 1:** The effect of *Chalk5* knockout on appearance quality, milling quality, and yield-related traits in two hybrid backgrounds.

Trait	LX03S/9311	LX03S/TC1	Huhan82S/9311	Huhan82S/TC1
PGWC (%)	51.24 ± 5.28	16.83 ± 2.97 **	13.3 ± 1.09	2.5 ± 1.24 **
DEC (%)	24.94 ± 3.06	4.34 ± 0.91 **	3.25 ± 1.27	0.63 ± 0.77 **
HMRR (%)	38.86 ± 5.97	40.02 ± 7.28	36.79 ± 6.21	38.44 ± 8.02
PN	8.51 ± 0.88	8.16 ± 0.74	7.84 ± 1.09	8.16 ± 0.94
SN	146.39 ± 5.24	153.01 ± 6.28	149.73 ± 10.22	141.48 ± 4.00
GN	114.27 ± 3.08	118.96 ± 4.88	126.41 ± 5.94	115.79 ± 5.23
SSR (%)	80.12 ± 5.25	79.63 ± 7.24	83.17 ± 5.28	80.60 ± 10.99
KGW (g)	30.06 ± 0.64	27.39 ± 0.77	29.79 ± 0.65	28.07 ± 0.93
GY (g)	38.21 ± 5.24	36.77 ± 2.06	40.27 ± 5.23	39.03 ± 6.66

LX03S/9311 is the F_1_ hybrid of LX03S and 9311, while LX03S/TC1 is the F_1_ hybrid of LX03S and the *Chalk5* knockout line TC1. Huhan82S/9311 is the F_1_ hybrid of Huhan82S and 9311, while Huhan82S/TC1 is the F_1_ hybrid of Huhan82S and the *Chalk5* knockout line TC1. PGWC, percentage of grains with chalkiness; DEC, chalkiness degree; HMRR, head milled rice rate; PN, panicle number; SN, spikelet number per panicle; GN, filled grain number per panicle; SSR, seed setting ratio; KGW, thousand grain weight; and GY, grain yield. ** represents significance at *p* < 0.01. Error bars represent the standard deviation for each line (n = 20 for PGWC, DEC, and HMRR; n = 10 for PN, SN, GN, SSR, KGW, and GY).

## Data Availability

The supporting data involved in this article are all original and can be provided upon request.

## Supplementary Materials

The following supporting information can be downloaded at https://www.mdpi.com/article/10.3390/biology13080617/s1. Supplementary File S1: amino Acid sequences of two knockout lines of *Chalk5* and 9311; Supplementary Figure S1: the appearance quality performance of two knockout lines of *Chalk5* and 9311; Supplementary Figure S2: the appearance and milling quality performance of two knockout lines of *Chalk5* and 9311 under high-temperature conditions; and Supplementary Table S1: the yield-related traits of two knockout lines of *Chalk5* and 9311.
