# Supplementary material for: Generation of Two-Line Restorer Line with Low Chalkiness Using Knockout of Chalk5 through CRISPR/Cas9 Editing
Source: Biology (Basel). 2024 Aug 15;13(8):617. doi: 10.3390/biology13080617 (PMC11351539; doi:10.3390/biology13080617)
Supplement: Supplementary file 1 [file biology-13-00617-s001.zip › biology-3129278-supplementary-figures.pdf]

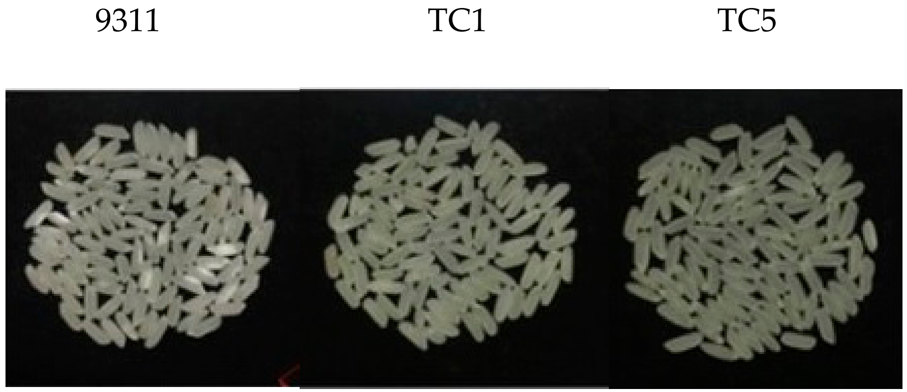

Supplementary Figure S1. Appearance quality performance of two knockout lines of *Chalk5* and 9311. The 9311 line represents the wild type, while TC1 and TC5 were two different homozygous *Chalk5* knockout lines in the T_1 generation.

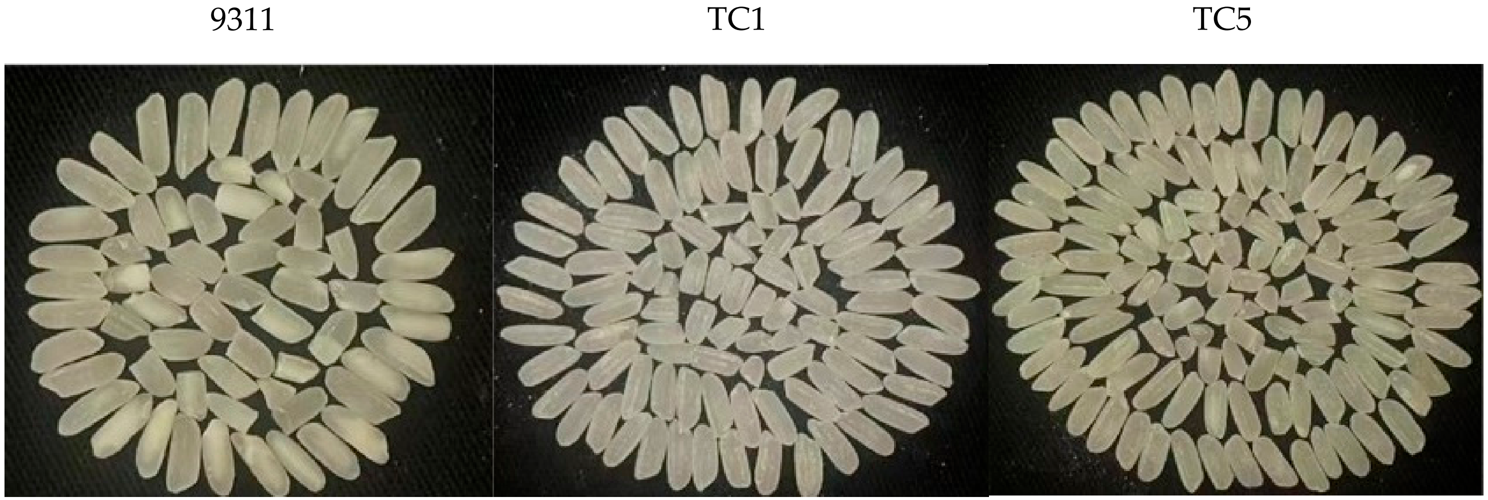

Supplementary Figure S2. Appearance and milling quality performances of two knockout lines of *Chalk5* and 9311 under high-temperature conditions.

The 9311 line represents the wild type, while TC1 and TC5 were two different homozygous *Chalk5* knockout lines in the T_1 generation.
