# Supplementary material for: Generation of Two-Line Restorer Line with Low Chalkiness Using Knockout of Chalk5 through CRISPR/Cas9 Editing
Source: Biology (Basel). 2024 Aug 15;13(8):617. doi: 10.3390/biology13080617 (PMC11351539; doi:10.3390/biology13080617)
Supplement: Supplementary file 1 [file biology-13-00617-s001.zip › biology-3129278-Supplymentary file S1.pdf]

Supplementary file S1. Amino Acid sequences of two knock-out lines of *Chalk5* and 9311.
>9311
MALIGTVAAEVLIPLAAVIGILFAVLQWYMVSRVAVPPHDGVGGAGKVERESDGGDGDG
DGVDDEEDGVDYRGVEARCAEIQHAISVGATSFLMTEYKYLGAFMAAFAAVIFVSLGSVG
RFSTSTEPCPYDAARRCRPALANAAFTAAAFLLGATTSVVSGYLGMRVATFANARTALEA
RRGIGRAFAVAFRSGAAMGFLLASSALLVLFAAVNAFGLYYGDDWGGLYEAITGYGLGGS
SMALFGRVGGGIYTKAADVGADLVGKVERNIPEDDPRNPAVIADNVGDNVGDIAGMGSD
LFGSYAESSCAALFVASISSFGADHDFAAMMYPLLVSAAGIVACAATTLVATDAGELGAA
DEVAPALKRQILISTVLMTAAVAAVTFLSLPRSFTLFDFGERKLVKNWHLFICVSAGLWAG
LVIGYVTEYFTSNAYGPVQTVAQSCRTGAATNVIFGLAVGYKSVIVPIFAIAGAIYASFRLAA
MYGIALAALGMLSTIATGLTIDAYGPISDNAGGIAEMAGMPRRVRERTDALDAAGNTTAA
IGKGFAIGSAALVSLALFGAYVSRAGIRTVNVVSPRVFVGLLAGAMLPYWFSAMTMRSVGS
AALRMVEEVRRQFDEIPGLAEGLAAPDYATCVRISTDASLREMVAPGALVMASPLVAGTL
FGVEALAGLLAGALVSGVQVAISASNSGGAWDNAKKYIEAGATEEARSLGPKGSEAHKA
AVIGDTIGDPLKDTSGPSLNILVKLMAVEALVFAPFFAAHGGIVFNHL*

>TC1
MALIGTVAAEVLIPLAAVIGILFAVLQWYMVSRVAVPPHDGVGGAGKVERESDGGDGDGD
GVDDEEDGVDYRGVEARCAEIQHAISVGATSFLMTEYK*

>TC5
MALIGTVAAEVLIPLAAVIGILFAVLQWYMVSRVAVPPHDGVGGAGKVERESDGGDGDGD
GVDDEEDGVDYRGVEARCAEIQHAISVGATSFLMTEYKYPGRVHGGVRGGHLRLAGLRG
AVLHVDGAVPVRRGEAVPPGAGERGVHRGGVPPRRHHLGGLRLPRDAGGDVREREDGA
GGSPRDRAGVRGGVQVGRRHGVPAGVERAAGAVRRRERVRALLRRRLGRAVRGDHRVR
ARGVVHGAVRPRRRRDLHQGGRRRRRPRRQGGAQHPRG*
